# The History and Clinical Appearances of Syphilis, with Especial Reference to the Dangers Arising from Its Oral Manifestations

**Published:** 1894-07

**Authors:** Charles M. Whitney

**Affiliations:** Boston


### THE HISTORY AND CLINICAL APPEARANCES OF SYPHILIS, WITH ESPECIAL REFERENCE TO THE DANGERS ARISING FROM ITS ORAL MANIFESTATIONS.[1]

BY CHARLES M. WHITNEY, M.D.,[2] BOSTON.

Syphilis may be defined as a constitutional disease, contagious in character, chronic in course, and capable of transmission by heredity and by the inoculation on any portion of the body of an indefinite substance known as the syphilitic virus.

The name itself, possessing a peculiar significance,—a companion of swine,—was first applied to the disease in a mythical poem written in 1521 by a Veronese physician, one Hieronymus Fracastorius, who describes a mysterious complaint with which a herdsman called Syphilus was afflicted by the god Apollo as a punishment for paying divine homage to King Alkithous, instead of to himself.

In attempting to locate the starting-point of this now widely prevalent and destructive disease much time and labor have been spent. Certain exostoses and other changes found in prehistoric bones have been accepted by some as evidences of the existence of the disease at that period. By others descriptions in Chinese treatises written 2000 B.C. have been considered typical of

---

[1] Read before Harvard Odontological Society, January 25, 1894.

[2] Surgeon to the Genito-Urinary Department of Boston Dispensary.

syphilis. By others still, passages in the sacred writings of the Hebrews have received the same interpretation. Although there is no doubt that some form of local venereal disease existed as far back as literature itself, there are many objections to accepting any of these theories as to its origin.

Until the latter part of the fifteenth century the evidence of the existence of this disease is vague and unsatisfactory. Four hundred years ago, in the year 1494, Charles the Eighth of France was carrying on a campaign in Italy, and during the winter of 1494–95 was besieging Naples. It was at this time that an epidemic of a then new disease occurred among the soldiers, alarming in extent and destructive in effect. This, then, is the first known outbreak of the disease in question, and it is to Marcellus Cumanus, a Venetian army surgeon, that we owe the first description of it.

Many ingenious theories were devised to account for this sudden uprising of the disease. By some the corruption of the atmosphere on account of the prevalence of severe floods in Italy in the year 1494, by others the influence of the stars, were assigned as causes.

Several years later attempts were made to prove that the disease was imported from America by Columbus and his men. A statement by an Andalusian surgeon, Ruy Diaz de Isla, written in 1512, that he had treated cases of this disease among the men from the ships of Columbus before they landed, has been chiefly used as an argument in favor of this view.

But, inasmuch as Columbus landed at Barcelona in April, 1493, it is remarkable, if this view be correct, that the disease did not originate there instead of at Naples. The explanation most generally accepted at the present day is that the disease had existed in a mild form throughout Europe, and at the period mentioned the aggregation in one place of eight thousand men leading the loosest of lives, aided possibly by the debilitating effects of the peculiar atmospheric condition, had rendered circumstances peculiarly favorable to a general outbreak of the disease.

From this time syphilis rapidly spread through Europe, being extremely severe in character for the first ten years, and later assuming a milder form not far different from the form seen at the present day.

As new countries have been discovered and settled, this disease has kept pace with advancing civilization, until to-day no country is free from its destructive effects save Iceland and certain portions of Central Africa.

To acquire syphilis, two factors are necessary,—the virus and an abraded surface of skin or mucous membrane through which it may enter the system. The nature of this virus has received careful investigation at the hands of bacteriologists, but as yet with unsatisfactory results.

In 1884, Lustgarten described a bacillus, resembling closely the tubercle bacillus, which he had constantly found in syphilitic lesions. Other observers have found this bacillus, but the question is still undecided. The great drawback to experimental research in this direction is that animals are not subject to this disease, and inoculation of cultures cannot be made. It seems extremely probable that some form of micro-organism is present which enters the circulation, and that the resulting symptoms are produced by it or the products from it. Although the disease is most frequently acquired during sexual intercourse, this is by no means the only way, other lesions than those upon the genitals being quite as contagious and more dangerous from the lack of knowledge concerning them.

For the sake of convenience we divide the course of syphilis into three periods,—primary, secondary, and tertiary. Contact of the virus with a broken epithelial surface having occurred, absorption takes place through the lymphatic circulation, and the original abrasion may entirely heal. We have now the first peculiarity of this disease, a period of incubation. After about three weeks there appears at the point of infection the first symptom of syphilis, the initial lesion, or, as it is also termed, the chancre.

Presenting frequently a simple red, elevated spot, it slowly becomes larger; and varying with its location we have another of its characteristics, a dense cellular infiltration about its circumference, which gives a firm, indurated, board-like feeling to the surrounding structures. The softer the tissues in which this is situated, the more marked this becomes. In certain situations—for example, the finger—it may be entirely absent.

Gradually increasing till an ulcerated surface of varying size is formed, differing in appearance according to the locality involved, this shows no tendency towards healing, but remains stationary after a certain size is reached. We now have a second period of incubation averaging about six weeks, when the secondary stage of the disease is reached.

During this period of incubation evidences of the lymphatic absorption of the poison can be found in the glands nearest to the primary sore,—upon the genitalia, those of the groin, upon the fingers, the epitrochlear, and axillary, upon the lip, the submaxillary.

These are enlarged, and present the same hard—usually painless—condition. The second stage is ushered in by feverish sensations, sore throat, pain in the bones, headache, and feelings of general malaise. Closely following these there occurs upon the surface of the body an eruption of rose-colored spots or blotches, closely resembling that of measles. This may cover the whole or only a portion of the body, is devoid of any subjective sensations, produces no discomfort whatever. It lasts two or three weeks, and disappears with a slight scaling of the skin. At the same time there occurs a general enlargement of the lymphatic glands throughout the body. The sore throat gradually disappears, the headache diminishes in severity, the initial lesion may shortly heal, and the patient may be lulled into a sense of false security.

Wide variations are frequently present: the sore throat and headache may persist, or the secondary rash may be replaced by others of a more severe character, but in the majority of cases the early secondary symptoms pursue the course outlined above. There now recurs a third period of quiescence, presenting no such regularity in course as the previous ones.

In rare cases nothing more may be seen for years, but the disease eventually asserts itself in some form. Usually closely following the disappearance of the secondary rash certain changes occur in the hair-bulbs, the nutrition of the hair becomes impaired, and baldness may result. Not infrequently the eyebrows or beard fall out. Later other lesions may be present on the skin,—papular eruptions, pustules disappearing by the formation of crusts, so that the whole body may be irregularly covered with them. To describe in detail even a small part of the various skin lesions would be outside of the limits of this paper, but it will suffice to say that they are wonderfully varied both in character and degree. The nails become brittle, crack, and may fall out. Coincident with these changes there occur certain characteristic lesions in the mouth and throat, to which I wish presently to recur in some detail.

As the disease progresses there appear the especial lesions of the tertiary stage. These develop usually after a lapse of two to five years, and are characterized especially by a tendency to involve the deeper and more important structures, viscera and organs of special sense. There occurs in this stage the formation of circumscribed cellular infiltrations,—a form of new growths known as gummata, which undergo a process of degeneration, producing deep ulcerations and their effects. In the relentless course of this disease no organ or structure is spared: deep ulcerations occur in the various mucous membranes; the voice may be impaired or lost; the nasal bones may be destroyed and the organ replaced by a hideous hole in the face; the hearing and sight may alike be destroyed from lesions of these organs. The brain and spinal cord are frequently affected, and paralysis, insanity, and death may result. The coats of the blood-vessels may become the seat of changes leading to the formation of large aneurismal growths, alike destroying life. Frightful deformities, interfering alike with function and personal beauty, actual destruction of life, may result before this direful disease has spent its force.

Happily, to view for a moment the reverse side of the picture, but few of these dreadful calamities are seen in properly-treated cases.

Nor are its effects limited to the person affected; children that are born of syphilitic parents may bear traces of the ravages of the disease in deformities of face or form, or loss of some important function. In them there is no regular succession of stages, but there are present the later and more serious lesions already alluded to. The one symptom which is of interest to this Society is a peculiarity of the permanent teeth which is doubtless perfectly familiar to all of you, consisting in a notching of the central upper incisors, known as Hutchinson teeth, from the English syphilographer who first called attention to them.

Leaving, then, with this brief sketch of its symptoms and its results, the subject of general syphilis, I wish to call your attention to certain peculiarities of the disease which are found in the mouth. The lesions in this region are second to none in importance in the whole body. Among the earliest to appear, the most obstinate and rebellious to treatment, and most apt to recur, of any of the symptoms, they are, in the early stages at least, the most contagious. There can be no doubt that more cases of extra-genital syphilis are acquired from them than from any local lesions elsewhere upon the body. It is this fact which gives to syphilis its chief interest to the dental profession and constitutes its chief danger. Patients who are perfectly aware of the danger from the primary lesion are ignorant of the risk of oral contamination, and spread the disease among the innocent.

Syphilis as seen in the mouth and throat presents five forms: the primary lesion; erythema,—that is, a localized congestion or redness; the mucous patch; gummata; ulcerations, superficial and deep, and their resulting cicatrices.

The primary lesion, most frequent upon the lower lip, may be found anywhere in this region, even as far back as the tonsil. It varies in appearance with its situation, being often a grayish ulcerated surface, again a hard, painless bunch, its usual form upon the lip. The erythema or congestion occurs at the same time as the secondary rash, and consists in a diffuse redness of the posterior parts of the mouth, chiefly on the soft palate and uvula, often having a butterfly arrangement, usually sharply defined from apparently normal mucous membrane at the junction of the hard and soft palate.

We occasionally see, however, many small red areas scattered over the roof of the mouth and on the cheeks, corresponding to the blotches upon the skin. This form lasts but a short time, either completely disappearing or changing to the third and most important form of oral syphilis,—the mucous patch, as it is termed. In number these patches may be one or many, and consist in whitish spots of varying size and shape, closely resembling the ordinary "canker spots" so often seen. Their usual situation is upon the inside of the lips and cheeks, upon the gums and tongue, especially when it comes in contact with the sharp edge of a tooth. When these patches exist in the corner of the mouth very troublesome cracks may result. They are somewhat sensitive to both heat and cold. Appearing first eight to twelve weeks after the primary sore, they are the most important of any of the lesions in this region.

Again and again, when the disease is supposed to be under control, and when no symptoms exist elsewhere upon the body, these mucous patches appear. Present alike in early and late syphilis, they are a constant source of danger. They are extremely contagious, and infect the saliva so that it, too, may convey the disease. Any local irritation favors an outbreak of these. They may change by a process of degeneration to superficial ulcerations.

Gummata are not frequent, but when present are found in the soft and hard palate and tongue especially. Their chief importance is in their terribly destructive action. Frequently in a few hours ulceration may occur, and a perforation or destruction of the palate result. The cicatrices resulting from these lesions have a very important diagnostic value.

From the foregoing description it will be seen that while these manifestations are often perfectly clear and distinctive of syphilis, it not infrequently happens that only a few scattered patches are present which bear a close resemblance to the common and harmless "canker spots." Being obviously, in most cases at least, unable to closely question the patient, how shall the dentist determine from physical signs alone one variety from the other? From the appearance of the local lesion itself it is often quite impossible to determine this, but, bearing in mind these general physical signs, he should look carefully for them.

Those accessible to ordinary inspection are: eruptions of various sorts; glandular enlargements, especially behind the ears and at the back of the neck; thinning of the hair, and especially of the eyebrows, with or without the presence of scabs in this location; and cracks or scars at the corners of the mouth and on the tongue. If any or all of these be present, it would afford strong presumptive evidence of the syphilitic nature of these patches. If these symptoms are all absent, he cannot hastily conclude that the affection is of an innocent nature.

The question naturally arises as to when these lesions cease to have contagious properties. It is the generally accepted opinion among syphilographers that late tertiary lesions are not contagious, some placing the time limit at three years after the primary sore, others at five. Although this may doubtless be true, yet no careful person would venture to expose himself even at a later period. The safe view to take is that any lesion occurring in the mouth at any period may be contagious, and to take precautions accordingly. In addition to these various lesions, the blood from a syphilitic patient possesses markedly contagious properties, a fact to be kept in mind in extracting teeth, and in other similar operative procedures.

From these various sources danger presents itself to the dentist in two ways. By contact of the saliva or secretion from these lesions upon an abraded spot in his hand a chancre may result, or by the deposition of the virus upon the various instruments, inoculation of other patients may occur.

The subject of extra-genital transmission of syphilis has received considerable attention of late years, and literature abounds in instances of it.

Dr. L. Duncan Bulkley, of New York, the foremost authority in this country upon the subject, has carefully and laboriously collected the evidence, and in his book, "Syphilis in the Innocent," shortly to appear, has presented a startling array of facts. He has also presented such facts as are of interest to the dental profession in a paper read before the New York Odontological Society, and published in INTERNATIONAL DENTAL JOURNAL for August and September, 1890.

It is to this, which should be read by every dentist, that I am indebted for many of the facts which I shall presently lay before you. The thought may very naturally occur to one unfamiliar with the subject, that if the method of infection is so common, why is it that we so rarely see it? why is it so uncommon to hear of an instance of it among dentists? The most reasonable explanation of this is to be found in the very natural diffidence which every individual who is the subject of syphilis, acquired innocently or otherwise, has to informing the community of the fact. It is to answer such criticism as this that I desire to lay before you the evidence that we possess on this subject,—the plain facts rather than any theories.

Dr. Bulkley has compiled a table of 9058 cases of extra-genital chancre, showing the locality in which they were situated.[1] Of these cases, the affection existed in the mouth and throat in 1504 cases, divided as follows: in the buccal cavity, 734 cases; upon the tonsils, 307; in the throat, 264; upon the tongue, 157; upon the gums, 42. Of the remaining cases there are 3249 which may reasonably be attributed, from their location, to infection from mouth lesions. These are divided as follows: upon the lip, 1810; the breast (from suckling infants), 1148; the chin, 146; the cheek, 145. This makes a total of 4753, nearly fifty per cent. of all the recorded cases which may have been produced by some form of oral syphilis. Besides these there are 462 cases where it existed upon the fingers and hand. These are facts collected from the practice of clinical observers who are competent judges of the nature of this disease.

Inasmuch as these recorded cases are but a small proportion of those which have occurred, who shall say that the danger is not worthy of careful consideration? Especially is this true in those branches of medicine which involve operative procedures about the mouth and throat.

Syphilis may be contracted from these various lesions, then, in two ways,—by direct contact or by contact with any article upon which the virus has been deposited. Of the first form instances are extremely common: surgeons and obstetricians have received the primary lesion upon the finger, and have in turn inoculated others before the nature of the trouble was recognized. Bites and tooth-wounds have also contributed their share, very many being reported.

A case which illustrates this method in a most vivid manner came under my observation two years ago. A young man of

---

[1] "System of Genito-Urinary Diseases. Syphilography, etc." Morrow, vol. ii. p. 69.

twenty-eight, strong and healthy, by occupation a bar-tender, in endeavoring to forcibly eject a man who was "fighting-drunk," struck him a blow upon the mouth and teeth, slightly wounding in so doing the knuckle of the middle finger of the right hand. The abrasion caused him no great inconvenience for about three weeks, when it became more tender and increased in size until an ulcerated surface the size of a ten-cent piece resulted. This refused to heal, in spite of vigorous treatment, and upon the occurrence of a perfectly typical rash the diagnosis was established. At this time he first came under my observation. He was put under treatment; the original lesion quickly healed, but a few mucous patches remained. He was cautioned regarding his domestic relations, and these directions were obeyed, except that he continued to kiss his wife on leaving and returning home. About three months later his wife came to the office with a chancre upon the lower lip and the evidences of constitutional syphilis.

From the original case another person was infected at the same time. In attempting to aid the first patient in ejecting this drunken syphilitic, a young man of thirty years was bitten upon the fingers, and he, too, developed a digital chancre and its resulting constitutional symptoms.

Another method of direct infection from lesions in the region under consideration is that of kissing. As seen by the cases already cited, the resulting chancre may be upon the lips or any part of the face, or the virus may be conveyed by the food or saliva to the deeper parts,—the tongue or tonsil.

They are by no means uncommon. I have seen six or seven of them, and other observers many more. The reported instances in which dentists have been infected in operating are by no means many, but the general impression among those paying special attention to the subject is that it is rather common. Bumstead states that he has known of several cases. Taylor makes a similar assertion. Dr. Bulkley reports four cases, one a personal experience reported by the dentist himself, another by Neunet, where infection occurred from the scratch of a tooth; another by Otis, which illustrates a peculiar method. A dentist received a chancre of the lip, caused by holding an instrument in his mouth while operating upon a patient who had a few mucous patches. The fourth, by Hutchinson, caused by a tooth-scratch. Besides those reported by Bulkley, Morrow gives a plate in his atlas showing a chancre upon the finger of a dentist. These recorded cases, and the strong presumption that many others have occurred, clearly show that care should be used.

Of instances of indirect contagion literature abounds. Any article which can possibly be brought in contact with a syphilitic lesion has produced the disease. There is apparently no end to the curious and interesting methods.

In 1577 an epidemic occurred at Brünn, by which one hundred and eighty people were inoculated by a cupping-glass in use at the bath. An ear specialist in Paris inoculated sixty people with a Eustachian catheter.

Pipes, musical instruments, forks, knives, spoons, children's toys such as trumpets and whistles, feeding-bottles, tooth-brushes, pencils,—in short, any article which can be transferred from mouth to mouth has carried the disease. An instance is on record of three workmen who were inoculated by biting pieces of thread from a ball which had been used in a like manner by a fourth, who was syphilitic. Public drinking-glasses, glass-blowing, coins, tacks and nails used in common by upholsterers and shoemakers, have all added to the cases. Tooth-transplantation has resulted in the development of a chancre.

Hundreds of interesting examples are found of indirect means, the mere mention of which would more than occupy the time allotted for this paper.

Of instances of inoculation from dental operations, Dr. Bulkley has recorded fifteen, the details of which may be found in his paper.

Such facts show most distinctly and clearly the care which should be taken in cleaning all instruments. The instruments and appliances used by the dental profession should receive the most painstaking attention.

Metallic instruments may be safe by boiling for half an hour in a 1 to 100 soda solution (a tablespoonful of washing soda to a quart of water). Others may be made tolerably safe by immersion in 1 to 10 creolin or pure carbolic acid. Mouth-mirrors seem to me particularly dangerous, and in a recognized case of syphilis a separate mirror should always be used. Towels and the like should be well boiled. In a word, surgical cleanliness will avoid the danger of indirect transmission of this disease.

Careful inspection of the fingers should not be neglected by the dentist, and any abrasion, particularly about the nail, should be brushed over with contractile collodion. In fresh tooth-wounds instant washing and the application of 1 to 500 sublimate solution or 1 to 10 carbolic solution should be resorted to. By these various means comparative immunity may be had from this insidious disease and its far-reaching effects prevented.

In conclusion, I desire to express my thanks to this Society for the privilege of presenting this paper, and for the opportunity of reviewing such facts as science has clearly established in relation to this subject. It is only by concerted action on the part of the medical profession that a great danger to the public health can be lessened.

It is not possible to fully accomplish this until public sentiment is aroused to the fact that this disease is not alone one occurring among the immoral and unclean, to be discussed under the breath, but that it exists boldly in our midst among all classes and conditions, threatening health and even life itself in the ordinary business and social relations.

When medical men, aided by other learned professions, shall have the courage to describe the disease as it is, its frequency and its dangers, we may expect an irresistible public demand for measures which shall limit its spread in the community.

Until this is done the innocent must suffer with the guilty, and the yet unborn will pay the penalty for parents' ignorance. If this paper shall have served to recall to your minds these facts, its object will be fully accomplished.

[Seven hospital cases, showing oral lesions of this disease, were brought by Dr. Whitney to illustrate his paper.]

